# Complications in Laparoscopic Access in Standing Horses Using Cannula and Trocar Units Developed for Human Medicine

**DOI:** 10.3390/vetsci10010061

**Published:** 2023-01-15

**Authors:** Francisco José Vázquez, Arantza Vitoria, Javier Gómez-Arrue, Sara Fuente, Laura Barrachina, Ignacio de Blas, Antonio Romero

**Affiliations:** 1 Equine Surgery and Medicine Service, Veterinary Hospital (HVUZ), Universidad de Zaragoza, C/Miguel Servet 177, 50013 Zaragoza, Spain; 2 Department of Animal Pathology, Faculty of Veterinary, Universidad de Zaragoza, C/Miguel Servet 177, 50013 Zaragoza, Spain; 3 Minimally Invasive Techniques Research Unit (UTMI), Universidad de Zaragoza, C/Miguel Servet 177, 50013 Zaragoza, Spain; 4 Instituto Aragonés de Ciencias de la Salud (IACS), CIBA, Avenida San Juan Bosco 13, 50009 Zaragoza, Spain; 5 Department of Anatomy, Embryology and Animal Genetics, Faculty of Veterinary, Universidad de Zaragoza, C/Miguel Servet 177, 50013 Zaragoza, Spain; 6 Instituto Universitario de Investigación Mixto Agroalimentario de Aragón (IA2), Universidad de Zaragoza, C/Miguel Servet 177, 50013 Zaragoza, Spain

**Keywords:** horse, minimally invasive surgery, laparoscopy, laparoscopic access, complications, body condition, endotip cannula

## Abstract

**Simple Summary:**

Accessing the abdominal cavity is one of the most delicate moments of laparoscopic procedures in horses. Laparoscopic cannula and trocar units have been specifically developed for horses; however, in mixed veterinary practices (small and large animals) it can be advantageous to use instruments designed for human patients, as these are suitable for both small animals and horses. Nevertheless, the body condition of the horse might influence the effectiveness of such devices. The aim of this work is to compare the efficacy and associated complications of the use of different devices developed for human medicine when used for laparoscopic access in the standing horse, analysing also the influence of body condition on the type and incidence of observed complications. A retrospective study was carried out including cases of laparoscopic access in standing horses in which devices for human surgery were used. The results showed that laparoscopy devices designed for human medicine can be used for laparoscopic access in the standing horse, but high body condition could be a risk factor for the appearance of complications. In addition, the use of optical devices, in particular optical helical cannula, minimizes the appearance of these complications, especially in horses that are overweight (OW).

**Abstract:**

First cannulation is a critical manoeuvre in equine laparoscopy. This retrospective study aimed at the comparison of the frequency and type of complications detected when using different human laparoscopy devices for laparoscopic access in standing horses, and the influence of body condition in such complications. Forty-four procedures were included, and retrieved data comprised cannula insertion technique, body condition, and type and frequency of complications. Laparoscopic access techniques were classified into five groups: P: pneumoperitoneum created using Veress needle prior to cannulation; T: sharp trocar; D: direct access via surgical incision; V: Visiport optical trocar and H: optical helical cannula (OHC). In groups T, D, V and H, access was achieved without prior induction of pneumoperitoneum. Complications were registered in 13/44 procedures, of which retroperitoneal insufflation was the most common (6/13). Statistically significant association was found between the complication incidence and the type of access, with group D showing the highest complication frequency (80%) and group H the lowest frequency (0%). The majority of complications (9/13) were observed in overweight horses. We conclude that devices designed for human patients can be used for laparoscopic access in standing horses, with the use of OHC minimizing the appearance of complications, especially in overweight horses with OW.

## 1. Introduction

Laparoscopic surgery is a common procedure in a growing number of equine hospitals [1]. Many of the surgical procedures in horses can be performed in the standing horse, avoiding the need for general anaesthesia and its associated complications and costs [2]. The insertion of the primary cannula is the most critical step in laparoscopic surgery. Several complications can occur at this stage, including the impossibility of gaining access to the abdominal cavity [3,4,5,6]. There are many commercial devices developed to perform laparoscopic access, either disposable or re-usable. Many of these devices are designed to be used in human medicine or in the ventral abdomen of the horse under general anaesthesia in dorsal recumbency [5,7,8,9,10,11,12]. Because of the cost of specific laparoscopic devices, it is important in veterinary hospitals with a mixed clientele of small and large animals to be able to use the same equipment for both small animals and horses. In this case, systems designed for human patients have the advantage of being suitable for different kinds of patients [13,14,15].

In laparoscopic surgery in the standing horse, the insertion of the primary cannula is performed through the flanks [6,16,17]. The abdominal wall in this area is much thicker than in the horse’s ventral midline or than in human patients. Furthermore, the peritoneum in the flank area is tougher and easier to separate from the retroperitoneal fat, compared to the ventral abdomen or the caudal thorax [4,5,7]. This anatomical feature can turn into the trocar pushing the peritoneum away from the abdominal wall, thus creating a “tent” without puncturing it. Therefore, several authors suggest performing the insertion of the primary cannula in the standing horse in the 17th intercostal space, even though this location may limit cannula positioning during surgery. In this intercostal area, the peritoneum is highly adhered to the abdominal wall, preventing the ‘tenting’ effect described above [5]. All of these distinctive features of the equine anatomy could be accentuated in overweight (OW) horses.

However, to the best of our knowledge, there is only one study retrospectively assessing different methods for laparoscopic cannulation in standing horses [18], but this study did not include devices such as optical helical cannula (OHC) or the possible influence of the horse body condition. Thus, the objective of this study was to compare the technical success and the associated complications of different techniques for the insertion of the primary cannula in laparoscopic surgery in the standing horse, by using devices designed for human patients and considering the possible influence of the horse’s body condition on the occurrence of complications. Our initial hypothesis was that, when using devices designed for human patients, performing laparoscopic access in the standing horse using systems with visual control (optical cannula) and in horses with a body condition score lower than six would result in fewer complications during primary cannula insertion.

## 2. Materials and Methods

### 2.1. Study Design

We conducted a retrospective study analysing the records of laparoscopic surgeries performed on standing horses during 5 years at the Equine Surgery and Medicine Service of the Veterinary Hospital of the University of Zaragoza (HVUZ). Inclusion criteria included laparoscopic surgeries performed in standing horses and by the same primary team of surgeons using human medicine designed devices for primary laparoscopic access.

Records from client-owned equine patients and from experimental horses undergoing laparoscopic surgery as part of other studies unrelated to this work were used. Laparoscopic procedures were not performed for the sole purpose of the present study, and all procedures involving experimental horses were carried out under Project Licence PI31/07, approved by the Ethics Committee for Animal Experiments from the University of Zaragoza and according to the Spanish Policy for Animal Protection RD53/2013, which transposes the European Union Directive 2010/63 on the protection of animals used for experimental and other scientific purposes.

The records were classified in five groups based on the technique used for the insertion of the primary cannula, as will be detailed below. Additional analyses were performed by grouping laparoscopic procedures in which optical vs. non-optical devices were used, i.e., with or without visual control, by placing a laparoscope inside the cannula during access or not. For each case, the recorded data included age; sex; breed; weight; body condition score; cannulation technique; laparoscopic devices employed; successful access to the peritoneal cavity; associated complications. As stated in the inclusion criteria, all horses underwent laparoscopy at the same hospital, by the same primary surgeon, following the same surgical and preoperative assessment protocols. Harmonization of these conditions would reduce the effect of other possible operator-dependent variables that were not analysed in this study.

### 2.2. Body Condition Assessment

The assessment of the body condition score of all horses was always carried out by the same person (SF), using the Henneke Index [19]. All horses with an index value equal to or higher than 6 were considered as OW horses.

### 2.3. Perioperative

All horses received prophylactic perioperative antimicrobials (sodium penicillin: 22,000 UI/kg IV QID, and gentamicin 6.6 mg/kg IV SID) and flunixin meglumine (1.1 mg/kg BID) or phenylbutazone (2.2 mg/kg IV or PO BID) prior to the surgical procedure and for at least 48 h after surgery.

In all cases, solid food was withheld for 18–36 h before surgery and free access to water was given until 2 h before the procedure. All horses were handled according to their temperament to allow the laparoscopic procedure to be performed with the horse standing and restrained in a stock. The administration of neuroleptoanalgesic drugs was performed through a 12 G catheter secured in a jugular vein. All horses were sedated with an IV bolus of romifidine (0.03–0.08 mg/kg) and butorphanol (0.02 mg/kg). Local anaesthesia was administered subcutaneously and through the muscular layer of the abdominal wall at the sites designated for cannula insertion (10–20 mL of 2% lidocaine). During the procedure, additional boluses of romifidine and butorphanol were administered at the anaesthesiologist’s judgement.

Preoperative transrectal examination and flank ultrasonography were performed to discard the presence at the cannulation site of the caudal margin of the spleen, distended intestinal loops, or adhesions. Following the protocols and assignment of tasks in our service, these determinations were always carried out by the same veterinarian (AV).

The selected place for the primary cannula insertion was prepared by clipping a 35 cm × 35 cm area in the paralumbar fossa and aseptically preparing for surgery. Primary cannula insertion was at equidistant point between the last rib and the most ventral part of the coxal tuberosity, above the prominence of the internal oblique muscle. All cannula insertions were performed by same surgeon (FJV), with more than 3 years’ experience in laparoscopic surgery of horses and large animals and with the use of these devices in experimental animals. In all cases, the first laparoscopic access was in the left flank, except in four individuals in which the primary cannula insertion was performed in the right flank.

A 57 cm working length, 0° and 10 mm endoscope (Hopkins 62032AP, Karl Storz GmbH & Co. KG, Tuttlingen, Germany) and a laparoscopic CO_2_ insufflator (SCB Electronic Endoflator 264305 20, Karl Storz GmbH & Co. KG, Tuttlingen, Germany) programmed at a maximum pressure rate of 15 mm Hg were used for all cases.

### 2.4. Cannula Insertion Technique

#### 2.4.1. Group P

**Figure 1:**
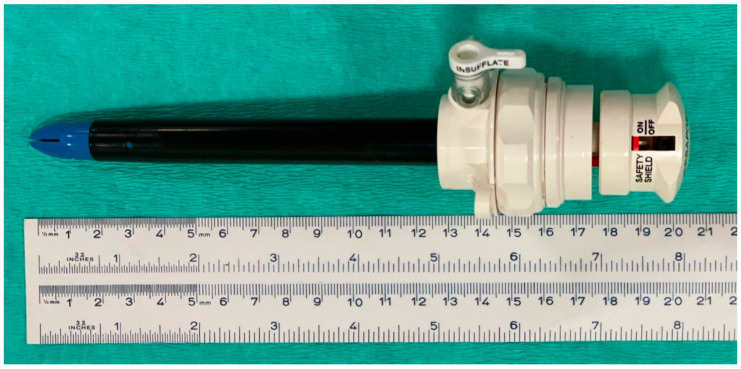
Device used in Group P after the induction of pneumoperitoneum: laparoscopic disposable cannula (12 mm diameter, 11 cm length). The cannula had a shielded sharp trocar of 13 cm.

Before the insertion of the primary cannula, the pneumoperitoneum was induced by insufflating CO_2_ through a 15 cm long and 14 G diameter disposable Veress needle (Surgineedle™ 150 mm Long Needle, Covidien AG, Mansfield, MA, USA). The needle was inserted through a small surgical incision in the skin with a slight ventro-caudal tilt. This device has a blunt obturator that is automatically released upon entry of the needle tip into the peritoneal cavity, which prevents the perforation of abdominal viscera. Adequate placement of the Veress needle inside the abdominal cavity was confirmed when a drop of saline solution was aspirated (hanging drop technique) into the abdomen or as soon as negative pressure (−1 to −5 mm Hg) was recorded on the insufflator manometer. CO_2_ was then insufflated through the needle until reaching the desired intra-abdominal pressure (maximum 15 mm Hg). At that moment, the needle was removed, and the skin incision extended to accommodate a laparoscopic disposable cannula (12 mm diameter, 11 cm length) with a valve to allow the insertion of instruments from 5 to 12 mm width (Versaport™ Long Sleeve, Covidien AG, Mansfield, MA, USA). The cannula had a shielded sharp trocar of 13 cm length (Versaport™ Plus V^2^ RT 12 mm Obturator) (Figure 1).

#### 2.4.2. Group T

**Figure 2:**
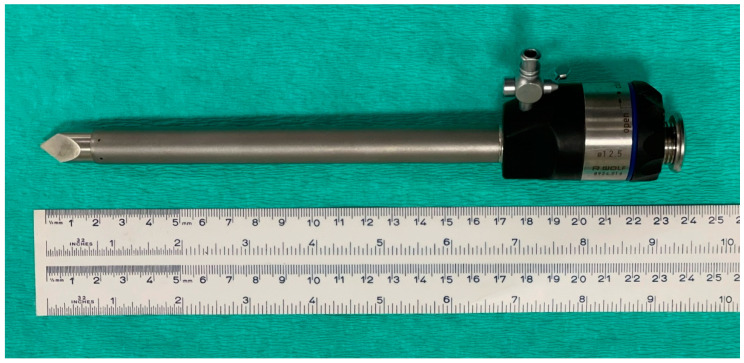
Device used in Group T. Laparoscopic cannula (12.5 mm diameter, 15 cm length) with a pyramidal tip trocar of 17 cm.

Pneumoperitoneum was not induced before initial cannula insertion. The skin was incised to insert a metal laparoscopic cannula (12.5 mm diameter, 15 cm length) with a pyramidal tip trocar of 13 cm (Ref. 8924.016, Richard Wolf GmbH, Knittlingen, Germany) (Figure 2). The cannula was directed slightly caudo-ventrally, pushing firmly with the palm of the hand to go through the abdominal wall and gain access into peritoneal cavity.

#### 2.4.3. Group D

**Figure 3:**
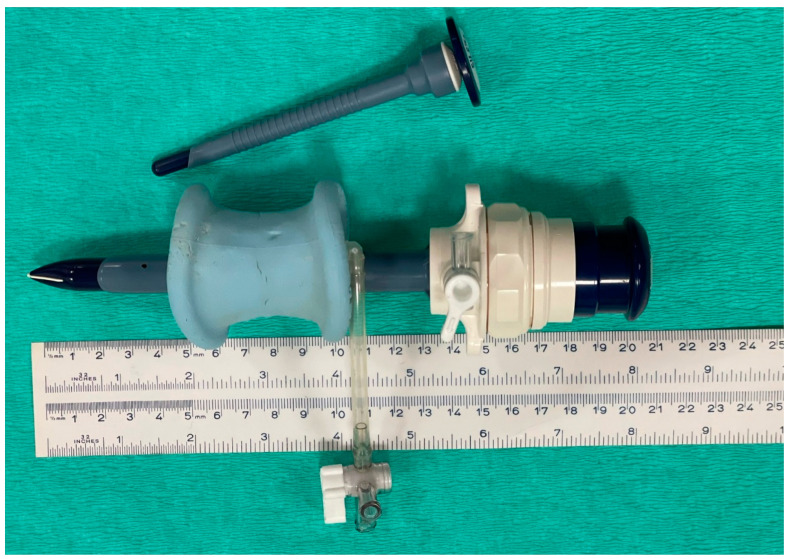
Device used in Group D. Single-port access system was used for one of the two methods included in group D: conformable device (blue foam) that adapts to the incision and seals it, presenting three orifices, one to insert a cannula (12 mm diameter, 11 cm length) for the endoscope and two for 5 mm instruments (at the top of the picture, not inserted in the foam).

Pneumoperitoneum was not induced before primary cannula insertion. A 4 cm long surgical incision was performed in the flank. The different muscles were dissected following a mesh pattern until the peritoneum was reached. At this point, a cannula was inserted to puncture the peritoneum and reach the abdominal cavity. Two different cannulas were used in different horses, depending on the procedure: either a laparoscopic disposable cannula (12 mm diameter, 11 cm length) with a blunt trocar of 13 cm (Versaport™ Sleeve, Covidien AG, Mansfield, MA, USA) or a single-port access system (SILS™ Port 12 mm, Covidien AG, Mansfield, MA, USA). The SILS system consisted of a conformable device that adapts to the incision and seals it, presenting three orifices to insert cannulas with a blunt trocar, one for the endoscope (12 mm diameter) and two for the instruments (5 mm diameter) (Figure 3).

#### 2.4.4. Group V

**Figure 4:**
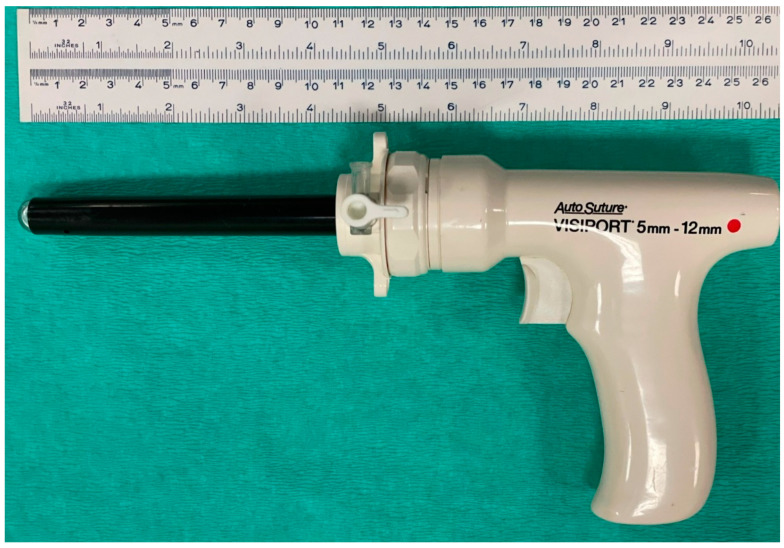
Device used in Group V. Disposable optic Visiport™ device inserted through a laparoscopic cannula (12 mm diameter, 11 cm length).

Pneumoperitoneum was not induced before initial cannula insertion. A 15 mm surgical incision was performed in the skin of the flank. Through that incision, a disposable Visiport™ (Covidien AG, Mansfield, MA, USA) device was inserted. This is a gun-like device that is inserted through a laparoscopic cannula (12 mm, 11 cm length) (Figure 4). The device incorporates a protected blade in the tip, which is activated from a trigger in the handle. This blade acts like a whip, which cuts the muscle fibres. The pressure applied during the procedure along with the incisions made by the blade allows access to the abdominal cavity. In addition, a 0° 10 mm endoscope can be placed inside the transparent tip of the device, for visual control of the access procedure.

#### 2.4.5. Group H

**Figure 5:**
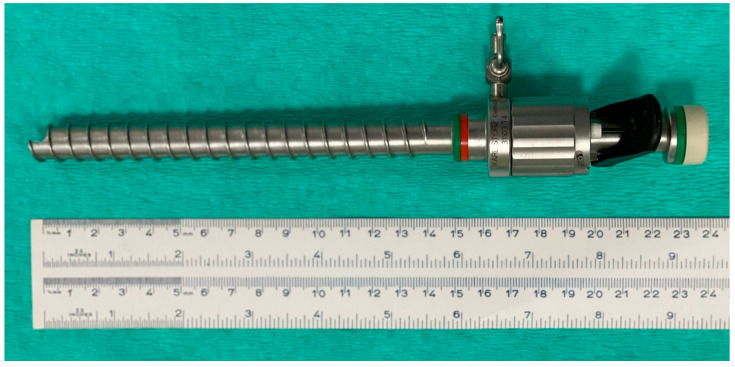
Device used in Group H. Optical helical cannula (12 mm, 15 cm length).

Pneumoperitoneum was not induced before initial cannula insertion. A 15 mm surgical incision was performed in the skin of the flank. Through this incision, an OHC (12 mm diameter, 15 cm length) was inserted (EndoTIP, Endoscopic Threaded Imaging Port, Karl Storz™) (Figure 5). This cannula allows placing an endoscope inside for visual control of the abdominal access. The exterior of the cannula is designed with a helical pattern that ends at the tip in a small prominence. When spinning the cannula clockwise while pressing the device slightly ventro-caudally, the prominence of the tip engages the different layers of the abdominal wall, separating them with no incision, while the helical outer pattern advances the cannula through the different tissues towards the abdominal cavity.

### 2.5. Statiscal Analysis

Descriptive statistics were performed using IBM SPSS 19.0 for Windows. Qualitative variables were described as absolute and relative frequencies, while quantitative variables were characterized in terms of mean, standard deviation, and range.

To determine if two qualitative variables were associated, the pertinent contingency table was generated, and the significance of the Pearson’s Chi-squared test calculated. In the cases in which Pearson’s Chi-Squared test was invalid (more than 20% of the expected values under 5), the significances of Fisher’s exact tests were calculated (in the case of 2 × 2 tables) or the likelihood ratio test (for the rest of the cases). Lastly, to determine which categories were associated, we calculated the adjusted standardized residuals, and the observed values were considered to be significantly higher when the value was higher than 1.96 and significantly lower when they were lower than −1.96.

In all cases, the α error was fixed at 0.05 (implying a confidence interval of 95%).

## 3. Results

Forty-one horses undergoing 44 laparoscopic procedures were included, comprising 30 laparoscopies in clinical cases and 14 laparoscopies from an experimental study not related to this work. Three horses underwent more than one intervention: two of them because the first laparoscopic access was unsuccessful and the laparoscopy had to be postponed, and one mare who required two interventions because of the presented condition. The 30 clinical cases were operated for the following indications; 11 exploratory laparoscopies (two of which included intraoperative biopsies), 6 cryptorchidectomies and 6 ovariectomies each, 3 inguinal ring hernioplasties, 3 cases of nephrosplenic space ablations, and in one case of haemostasis of the testicular vasculature, to stop post-castration haemorrhage.

The distribution by sex and breed of the horses submitted to the 44 laparoscopies is shown in Table 1. Age ranged from 5 to 24 years (mean 14.2 ± 5.3). Body weights ranged from 309 to 609 kg (mean 487.57 ± 62.51). Based on the body condition score, 61.36% (27/44) of the horses that underwent laparoscopy were categorized as being OW. This proportion reached 83.33% in Purebred Spanish (PRE) horses.

The distribution of the 44 laparoscopic procedures according to the type of access is shown in Table 2.

### Type and Frequency of Complications According to the Method of Laparoscopic Access

Among the 44 laparoscopies reviewed, there was a total of 14 complications related to access to the abdominal cavity (i.e., placement of the primary laparoscopic portal) (31.81%). In one case, two complications occurred in the same laparoscopy (marked gas leak and puncture of the iliac circumflex artery), so the total number of procedures with complications was 13 out of 44 (29.55%).

Five different types of complications were detected. According to the laparoscopic access technique used, the results shown in Table 3 were recorded.

The complication frequency was significantly different according to the type of access (*p* < 0.001), with group H having a statistically lower complication frequency (0%, 0/18) and group D having a significantly higher frequency (80%, 4/5) than the rest.

In two of the 13 procedures with complications (15.38% of the cases with complications and 4.54% of the total number of laparoscopies), the surgery had to be postponed as a result of these complications. In these two cases, the complication recorded was peritoneal detachment from the abdominal wall (‘tent’ effect) and consequent retroperitoneal insufflation. Both cases were in Group P. The retroperitoneal insufflation forced postponement the laparoscopic procedure due to the impossibility of placing the laparoscopic portal.

In the rest of the procedures with complications during laparoscopic access (11 of 13, 84.61% of total procedures with complications), it was still possible to access the peritoneal cavity, and the procedure was completed despite the initial incidents. The 11 complications included 4 peritoneal separations (2 in Group P and 2 in Group V), in which the problem was quickly detected and the CO_2_ flow stopped until the peritoneum was perforated (using either the endoscope itself or laparoscopic scissors). Then, the cannula was conveniently repositioned inside the peritoneal cavity and the laparoscopic procedure continued.

In the other four procedures, the complication detected during access was a marked gas leakage, and all of them took place in Group D. Two of the gas leakage situations happened when using a blunt cannula, and the other two leakages occurred while using the SILS™ port device. In all cases, this complication was successfully managed by placing towel clamps and/or doing manual compression around the portal. The surgeries were completed, with the only issue being consuming considerably more CO_2_ than usual.

Another complication detected was the puncture of the spleen; this happened in two procedures. One of the spleen punctures occurred in Group P while creating the pneumoperitoneum prior to abdominal access. In this case the complication was detected right after placing the cannula and exploring the abdomen with the endoscope. In the second case, the spleen was punctured when using an acute pyramidal trocar without prior insufflation in Group T. In both Group P and Group T, cases the haemorrhage could be controlled by applying pressure with gauze through an accessory portal. In the Group P case, the puncture had a small diameter, as it was done with the Veress needle. In the second case, in spite of the higher invasiveness of the trocar used, only a small area of the dorsocaudal border of the spleen was lacerated. In both cases and after controlling the haemorrhage, the intervention could be completed with no other inconvenience than the time invested in haemostasis. The horses did not present any additional complications after surgery.

There was one case of perirenal puncture in Group T, with the cannula inserted retroperitoneal and near the kidney. We became aware of the complication when the endoscope was introduced after removing the trocar, and it was found to be lodged in the retroperitoneal fat, as the cannula placement and trajectory were too dorsal. Since no damage to the renal parenchyma was detected and CO_2_ had not been insufflated, the cannula could be successfully repositioned through a more ventral incision, and the intervention was completed without further complications. The horse showed no signs of complications after the procedure.

Puncture and bleeding of the iliac circumflex artery was recorded in one case in Group D, caused by the incision of the abdominal wall to place the SILS™ port device. Since this is an open technique, it was possible to control the haemorrhage by mass ligation of the bleeding area with USP 2/0 uncoated, monofilament poly-p-dioxanone absorbable material (Monoplus^®^, B Braun, SA, Barcelona, Spain).

Furthermore, cases were grouped into cases with visual control of laparoscopic access, and cases without visual control of entering the abdomen. Thus, in all cases included in groups P, T and D are techniques in which the cannula was placed without visual control (*n* = 19). In all remaining cases (*n* = 25), i.e., in groups V and H, laparoscopic access was performed with an optical cannula that allowed for visual control of the entry procedure (Table 2). Comparing the two categories, i.e., with or without optical cannula, complications were documented in 8% (2 out of 25) of laparoscopic accesses performed under visual control versus 57.9% (11 out of 19) accessed without visual control (Table 4). Thus, laparoscopic accesses under visual control had a significantly (*p* < 0.001) lower complication frequency compared to access systems without visual control.

From the 44 laparoscopies reviewed, 27 (61.36%) were performed on OW horses (body condition score ≥ 6). Although the proportion of OW horses in the different groups of access techniques ranged from 44 to 71.4%, no significant differences in the distribution of OW horses were observed (*p* = 0.811). Ten out of the 14 (71.4%) complications recorded occurred in OW horses. Since two of these complications occurred in the same procedure, the complication frequency for laparoscopic access in OW horses was 33.3% (9 of 27), which was higher compared to 23.5% (4 of 17) in the group of non-OW horses and 29.5% (13 of 44) in the total number of procedures. The most frequent complication in OW horses was peritoneal separation (5 of 10, 50%). Among the horses with no OW, the most frequently recorded complication was puncturing the spleen (2 of 4, 50%). The type of access that caused most of the complications in OW horses was direct access with the Hasson technique (Group D), in which four complications were recorded in three procedures (two simultaneous complications happened in one procedure). The only type of access without recorded complications in OW horses was when OHC were used (group H) (Table 3).

In group H (*n* = 18), no complications occurred (complication frequency 0%), while in the rest of the laparoscopic accesses with other modalities (*n* = 26), a total of 14 complications were recorded in 13 procedures (complication frequency 50%). Table 5 shows the complication frequencies when the types of laparoscopic access were stratified according to the body condition of the horses (OW versus non-OW). The complication frequency when using OHC was significantly lower (pX2 < 0.001 and pFisher = 0.001) than the general complication frequency in horses with OW and in the total horses studied. However, no significant differences were found (pFisher = 0.237) for the use of the OHC system against the rest of methods if horses were not OW (body condition score <6).

## 4. Discussion

Although there are longer laparoscopic devices specifically designed for large animals, devices commercialised for human medicine are a convenient option because of their availability and versatility for different species. In all the laparoscopic procedures revisited in this retrospective study, human medicine-designed devices were employed to gain laparoscopic access (i.e., placement of the primary cannula). Approximately one-third of these procedures presented some type of complication related to abdominal cavity access. However, none of these complications can be considered a major complication with serious repercussions for the horse, although in two cases it was necessary to postpone the procedure due to retroperitoneal insufflation that prevented placing the primary cannula. These results on complication frequencies and severity of complications do not differ greatly from those obtained in small animals [14]. However, due to obvious differences between species, our results should be compared with other studies in standing horses. Unfortunately, as shown in a recent review on laparoscopic complications in horses, not many investigations have specifically studied complications related to laparoscopic access in this species [20]. To our knowledge, there is only one study focusing on complications at the laparoscopic access stage in standing horses [18]. However, it was not the primary objective of that work to critically assess the use of human medical devices used in horses; it mainly evaluated devices that were developed for human patients. However, the EndoTip-type cannula (Group H of the present study) was not included, most likely because the study was conducted between 1997–2002 and prior to the initial documentation of the use of this device for horses [8,21]. In spite of some differences in the approach between previous and current studies, our general results are very similar to those observed by Desmaizières et al. [18].

The complications recorded in our study have also been described by other authors. Retroperitoneal insufflation is cited in several studies [3,4,18]. In laparoscopy in the standing horse, laparoscopic access is gained through the flanks [16,17], in which the thickness of the abdominal wall is much greater than in the ventral abdomen or in the human patient; on the other hand, the peritoneum is more resistant and detaches from the retroperitoneal more easily than the ventral part of the abdomen or in the caudal thoracic region [4,7,10]. This explains why some human devices with a length of 10 cm or less may fail to reach the abdominal cavity, thus either missing insufflation or creating a retroperitoneal insufflation if this misplacement is not detected. In fact, 66.6% of our retroperitoneal insufflations (four of six) occurred in group P, which can be attributed to the relatively short length of the Veress needle used. This complication occurred in 13.6% of the interventions in our study, being the most frequently recorded. This result is similar to that found in the work of Desmaizières et al. [18], in which retroperitoneal insufflation also was the most frequently observed complication, with an incidence of 15%, only slightly higher than in our study. This complication could be minimized by employing longer devices such as some of those currently available for its use in horses; however, we could not find any works that specifically evaluated the complication frequency of portal placement with these methods in horses [20].

Leakage of CO_2_ and/or subcutaneous emphysema are described in some studies [3,4,18]. This is a relatively frequent complication that is usually minor, with a rapid resolution. In general, horses are not particularly compromised by subcutaneous emphysema; however, one case was described in which the emphysema caused dehiscence of the laparoscopic portal suture [18]. Another case was reported in which severe lameness developed after laparoscopic cryptorchidectomy because of the significant subcutaneous emphysema that appeared within 24 h after surgery. In this case, the emphysema extended cranially up to the 12th–13th intercostal space and caudally up to below the medial aspect of the right stifle, resulting in marked lameness that resolved within two days with topical anti-inflammatory treatments around the incision [22]. In our study, we observed gas leakage around the cannula, but it was not accompanied by notable emphysema during the postoperative period. Gas leakage was the second most common complication in our study, with a 9.1% frequency. In a previous study [18], postoperative emphysema was more commonly observed (30%) and required up to 7 days to resolve. All of these complications detected in our study took place in Group D (four of four, 100%), likely due to the larger size of the incision required for this type of access, which facilitates CO_2_ escaping around the cannula. This circumstance would explain why subcutaneous emphysema is not observed in these cases, since the gas escapes directly to the environment.

Spleen puncture during laparoscopic access is also cited in several studies [3,4,18]. This complication has been mostly described when laparoscopic access is performed in the standing horse, although it can also appear in ventral approaches. Splenic haemorrhages are generally self-limiting and do not involve major complications other than difficulty of endoscopic visualization for few minutes. The incidence of this complication in our study was 4.54%, which was lower than the 10% reported by Desmaizières et al. [18]. The two splenic punctures recorded in our study both resolved without subsequent repercussions, even in the case in which a sharp pyramidal trocar (Group T) was used. It has been described that the use of this type of pyramidal trocar, especially if used without prior pneumoperitoneum, increases the possibility of injuring the hollow or parenchymal abdominal viscera, so the use of sharp trocars is recommended along with protective systems that are activated when crossing the abdominal wall and entering the peritoneal cavity [1,4,6,7,8,9,10].

The injury of abdominal wall vessels during laparoscopic access is reported in different works [3,4,18]. The respective vessels most commonly involved in flank access in the standing horse are the circumflex iliac vessels [3], which are close to the dorsal border of the internal oblique muscle of the abdomen [4]. If a puncture occurs, haemorrhage is usually controlled by ligation, electrocautery, or by applying pressure via cannula access with laparoscopic instruments, and the repercussions are usually limited to increased procedure time, impaired visibility of the surgical field by the presence of blood, and occasionally subcutaneous hematoma or hematoma of the rectus abdominis muscle sheath or hemoperitoneum [3,4]. Nevertheless, some authors warn that ligation of these vessels can be a complex and stressful procedure that may require a larger incision [4]. In our study, puncture of the circumflex iliac vessel occurred in one case (2.72%) with the Hasson technique (group D) when opening the abdominal wall, while the retrospective study of Desmaizières et al. did not report this complication [18].

Retroperitoneal perirenal placement of the cannula is a complication infrequently described in the literature, although it is cited in some reviews, which indeed recommended placing the first portal at or below the ventral part of the coxal tuberosity to avoid penetrating the retroperitoneal space in the perirenal area [10]. In our study, this complication only occurred in one case (2.7% incidence) and could be resolved without causing further complications.

Inadvertent puncturing of the gastrointestinal viscera is the most feared complications, due to the serious potential repercussions that it can generate [3,4,18]. This complication was not recorded in our study, likely because we adhered to the recommendations proposed to reduce the risk of this complication, including fasting, rectal palpation, and ultrasound scan of the area [3,4]. Desmaizières and colleagues report [18] intestinal puncture in two cases (5% incidence); in one case, the catheter used for peritoneal insufflation was introduced into the small colon, which was managed with antibiotic instillation only, while in the other case a laparotomy under general anaesthesia was required to resolve a small colon perforation created by the laparoscopic cannula.

Neither in our study nor in that of Desmaizières et al. [18] was a puncture of genitourinary organs recorded (kidney), not even in the case in which we had a retroperitoneal puncture in the perirenal space. This complication has been reported in some review papers [3,4,18]. It has been theorized that if the primary cannula is introduced too high through the flank, the kidney could be punctured [3]. Nevertheless, correctly choosing the entry site and directing the device caudally should be sufficient to avoid this complication.

Overall, our results show that the type and frequency of complications during laparoscopic access in the standing horse when using human medicine devices is similar to what has been described so far in the equine literature. Unfortunately, there are no works assessing laparoscopic access with special long devices for horses that can be used for comparison with our data.

Our results show significant differences in the frequency of complications among the different systems used for laparoscopic access in the standing horse. Methods based on visually controlled access (optical cannula) had the lowest complication frequency in our study. Based on human experience with these devices, we can hypothesize that the effectiveness of these devices may be explained by a variety of factors. First, their design can reduce the risk of peritoneal detachment and the subsequent “tent” effect, even if the devices are no longer than 15 cm [23,24,25,26]. In addition, direct, real-time visualization of the access may avoid complications associated with a blind access lacking visual control and help detect other complications, such as damage to abdominal wall vessels [25,27], retroperitoneal CO_2_ insufflation [23,25], or damage to intra-abdominal viscera [24,25,26,27,28].

In particular, OHC has the lowest complication frequency (0%), while direct access with the Hasson technique showed the highest one (80%). The OHC presents two main advantages that can explain the better results observed with this system: first, this system uses a dissection rather than incision approach, which results in less damage to the fasciae and vessels of the abdominal wall; and second, the OHC allows visual control of the entrance procedure, as described in numerous studies in human [23,24,25,26] and canine [14] medicine.

In the study by Desmaizières et al. [18], the only access method reported with 0% complications was also an entry under visual control. This method used a cannula in which an endoscope with a working channel was placed. Through this endoscope, surgical instruments were introduced to dissect the abdominal wall. This method presents important advantages but could not be included in our study because of its significant costs. The second method with the lowest complication frequency in the study of Desmaizeres et al. was again another method allowing visual control: the Visiport™ device, with a complication frequency of 7.3%. This device was also included in our study, more precisely in the group with the second lowest complication frequency (28.6%). Although the Visiport™ system is also an optically controlled method, it showed a higher complication frequency than the OHC (28.6% vs. 0%) in our study. This could be explained by the fact that Visiport length is only 11 cm compared to the 15 cm length of the OHC used in this study. The length difference might be the reason why the Visiport system has been shown as less effective in equine laparoscopic procedures compared to that observed in human medicine [25]. However, the Visiport system has even allowed flank access in larger animals, such as giraffe, in a postmortem assay [29].

The frequency of the complications reported in our study could be increased in horses with OW. Our results show that the majority of the 14 total complications recorded were indeed in OW horses (71.43%) and that the complication frequency was higher than in horses without OW, although this difference did not reach statistical significance. In OW horses, retroperitoneal insufflation was the most frequent complication (50%). It is challenging to compare our results in OW horses with those previously described by other authors, mainly because we only found one retrospective study about laparoscopic access in horses, and it did not include body condition as a parameter for analysis [18]. However, in human medicine, elevated body condition score is taken into account as adding technical challenges in laparoscopic interventions in obese patients [27,30]. Different investigators recommend that specific entry techniques are recommended with this type of patient to minimise access-related complications, including open entry or direct access [31] or OHC [32]. The latter observation is consistent with our results in OW horses, where OHC was the method of entry with the fewest complications.

This study has several limitations, such as the low number of laparoscopies and its retrospective, non-standardised and non-randomised nature. It was only after categorizing procedures into non-optical vs. optical devices that we were able to confirm that visual control of the laparoscopic access can reduce the incidence of complications at this stage of the procedure. Furthermore, minor complications may have been underreported as a result of the retrospective nature of the study. The analyses of the present study have focused on factors with a potential impact on the incidence of complications that may arise during laparoscopic access in the standing horse, namely the device/system used and the body condition of the horse. However, other factors (including surgeon’s experience with each device, and many others) may have influenced the occurrence of complications in the cases included in our study, and such variables should be taken into account in future prospective studies. Nonetheless, the results of this study provide the first information about potential complications when using human medicine devices to gain laparoscopic access in OW equine patients and can provide a basis to develop more standardized studies investigating this topic. For instance, it would be more relevant regarding the presented research question to ultrasonographically measure the body wall thickness rather than categorizing horses based on their body condition score. This way, the authors could potentially have presented a cut-off value for the thickness of the abdominal wall in the paralumbar fossa for each respective cannula and trocar unit.

## 5. Conclusions

In conclusion, devices designed for use in human medicine can be used for laparoscopic access in the standing horse. In these situations, high index of body condition is a risk factor that influences the appearance of complications. The use of the optical helical cannule minimizes the appearance of these complications, mainly in OW horses.

## Figures and Tables

**Table 1:** Breed and sex characteristics of the 41 horses subjected to 44 laparoscopies. In parentheses, percentage with respect to total laparoscopies.

Breed	Gelding	Stallion	Mare	Total
Crossbreed	18	1	12	31 (70.4%)
PRE		4	2	6 (13.6%)
CDE	1	3	1	5 (11.4%)
TB		1		1 (2.3%)
Arb		1		1 (2.3%)
Total	19 (43.2%)	10 (22.7%)	15 (34.3%)	44 (100%)

PRE: Purebred Spanish Horse, CDE: Spanish Sport Horse, TB: Thoroughbred, Arb: Arabian Purebred.

**Table 2:** Distribution of laparoscopic procedures between groups. In parentheses, percentage of horses with overweight (OW) in relation to the total group.

Group	Total	OW	Category	Total	OW
P	9	4 (44.5%)	Without visual control	19	10 (52.6%)
T	5	3 (60%)			
D	5	3 (60%)			
V	7	5 (71.4%)	With visual control	25	17 (68%)
H	18	12 (66.6%)			
Total	44	27 (61.4%)		44	27 (61.4%)

OW: overweight horses.

**Table 3:** Recorded complications in relation to the laparoscopic access method. In parentheses, number of horses without (N) or with overweight (OW).

Complication	P (*n* = 9)		T (*n* = 5)		D ^†^ (*n* = 5)		V (*n* = 7)		H (*n* = 18)		Total (*n* = 44)	
	N (5)	OW (4)	N (2)	OW (3)	N (2)	OW (3)	N (2)	OW (5)	N (6)	OW (12)	N (17)	OW (27)
Retroperitoneal insufflation	1	3	0	0	0	0	0	2	0	0	1	5
Excessive CO_2_ leakage	0	0	0	0	1	3 *	0	0	0	0	1	3
Spleen puncture	1	0	1	0	0	0	0	0	0	0	2	0
Perirenal cannulation	0	0	0	1	0	0	0	0	0	0	0	1
IC artery bleeding Total	0 2	0 3	0 1	0 1	0 1	1 ^†^ 4 ^†^	0	0	0	0	0	1 ^†^
							0	2	0	0	4	10 ^†^
Stratified complications frequency	40.0%	75%	50.0%	33.3%	50.0%	100% ^†^	0%	40.0%	0%	0%	23.5%	37.0% ^†^
Total complications [frequency] *	5 [55.6%]		2 [40.0%]		5 ^†^ [80.0% ^†^]		2 [28.6%]		0 [0%]		14 [31.8% ^†^]	

IC: Iliac circumflex. P: Group P, previous pneumoperitoneum with Veress needle. T: Group T, pyramidal trocar. D: Group D, direct access with Hasson technique. V: Group V, Visiport™. H: Group H, optical helical cannula. ^†^: In one case of group D, two different complications occurred in the same procedure.*: Significance of the likelihood ratio test, *p* < 0.001.

**Table 4:** Complications related to the visual control during access method.

Complication	Access without Visual Control ^†^, *n* = 19		Access with Visual Control, *n* = 25		Total, *n* = 44	
	N (*n* = 9)	OW (*n* = 10)	N (*n* = 8)	OW (*n* = 17)	N (*n* = 17)	OW (*n* = 27)
Retroperitoneal insufflation	1	3	0	2	1	5
Excessive CO_2_ leakage	0	3 ^†^	0	0	1	3 ^†^
Spleen puncture	2	0	0	0	2	0
Perirenal cannulation	0	1	0	0	0	1
IC artery bleeding	0	1 ^†^	0	0	0	1 ^†^
Total	3	8 ^†^	0	2	4	10 ^†^
Stratified complications frequency	33.3%	80.0% ^†^	0%	11.8%	23.5%	37.0% ^†^
Total complications [frequency] *	11 ^†^ [57.9% ^†^]		2 [8%]		[31.8% ^†^]	

IC: Iliac circumflex. ^†^: In one case of group D, two different complications occurred in the same procedure. *: Significance of the likelihood ratio test, *p* < 0.0013.2.

**Table 5:** Complication frequency in laparoscopic access between Group H (using optical helical cannula, OHC) versus the rest of the systems studied and the total of the study, comparing overweight (OW) horses with non-OW horses with a body condition score < 6.

Body Condition Index	Group H (OHC)	Other Groups	Total	P
≥6 (OW)	0.0% (0/12)	60.0% (9/15)	33.3% (9/27)	0.001 ^F^
<6	0.0% (0/6)	36.4% (4/11)	23.5% (4/17)	0.237 ^F^
Total	0.0% (0/18)	50.0% (13/26)	29.5% (13/44)	<0.001 ^X2^

^X2^: Significance according to Pearson’s Chi-square test. ^F^: Significance according to Fisher’s exact test.

## Data Availability

The original contributions presented in the study are included in the article/supplementary material; further inquiries can be directed to the corresponding author.
